# The Hedgehog Inhibitor Cyclopamine Reduces β-Catenin-Tcf Transcriptional Activity, Induces E-Cadherin Expression, and Reduces Invasion in Colorectal Cancer Cells

**DOI:** 10.3390/cancers7030867

**Published:** 2015-09-17

**Authors:** David Qualtrough, Phil Rees, Beverley Speight, Ann C. Williams, Christos Paraskeva

**Affiliations:** 1 Department of Biological, Biomedical & Analytical Sciences, University of the West of England, Faculty of Health and Applied Sciences, University of the West of England, Frenchay, Bristol BS16 1QY, UK; 2 School of Cellular & Molecular Medicine, University of Bristol, Medical Sciences Building, University Walk, Bristol BS8 1TD, UK; E-Mails: phil.p.rees@gmail.com (P.R.); bevspeight@yahoo.com (B.S.); ann.c.williams@bristol.ac.uk (A.C.W.); c.paraskeva@bristol.ac.uk (C.P.)

**Keywords:** colorectal cancer, Wnt signalling, hedgehog signalling, E-cadherin, cyclopamine, β-catenin/Tcf, EMT, invasion

## Abstract

Colorectal cancer is a major global health problem resulting in over 600,000 deaths world-wide every year with the majority of these due to metastatic disease. Wnt signalling, and more specifically β-catenin-related transcription, has been shown to drive both tumorigenesis and the metastatic process in colorectal neoplasia, yet its complex interactions with other key signalling pathways, such as hedgehog, remain to be elucidated. We have previously shown that the Hedgehog (HH) signalling pathway is active in cells from colorectal tumours, and that inhibition of the pathway with cyclopamine induces apoptosis. We now show that cyclopamine treatment reduces β-catenin related transcription in colorectal cancer cell lines, and that this effect can be reversed by addition of Sonic Hedgehog protein. We also show that cyclopamine concomitantly induces expression of the tumour suppressor and prognostic indicator E-cadherin. Consistent with a role for HH in regulating the invasive potential we show that cyclopamine reduces the expression of transcription factors (Slug, Snail and Twist) associated with the epithelial-mesenchymal transition and reduces the invasiveness of colorectal cancer cells *in vitro*. Taken together, these data show that pharmacological inhibition of the hedgehog pathway has therapeutic potential in the treatment of colorectal cancer.

## 1. Introduction

Colorectal cancer (CRC) is a major global health problem, with 1.36 million new cases being reported in 2012, and over 600,000 deaths due to the disease occurring the same year [1]. Incidence is steadily increasing, and it remains the second leading cause of cancer deaths in the United Kingdom [1].

The Wnt signalling pathway has been known for some time to provide the key driving force behind neoplastic changes in the colorectal epithelium, and also to play a key role in malignant progression [2,3]. Mutational loss of APC function leads to constitutive activation of the canonical Wnt pathway, by allowing cellular accumulation of β-catenin. Binding of β-catenin to members of the TCF/LEF family of transcription factors increases the expression of a range of target genes, with diverse cellular functions, contributing to several of the “hallmarks of cancer” [4]. The role of Wnt is, therefore, well established in colorectal cancer, but this understanding has yet to yield any fruitful therapeutic approaches, therefore we need to consider the influence of Wnt in the context of other signals.

Signalling via sonic and indian hedgehog (SHH and IHH) proteins has been shown to be vital for intestinal development, but the precise role of these signals in tissue homeostasis and neoplasia remains unclear [5].

We were the first to show that the components of the hedgehog (HH) signalling pathway are expressed in cells derived from both colorectal adenomas (benign) and carcinomas (malignant) [6]. Both the mRNA and protein was detected for both SHH and IHH, as well as the receptor PATCHED1 (PTCH1) and downstream signalling components smoothened (SMOH) and GLI. These data suggested the possibility of autocrine HH signalling in cells from both benign colorectal adenomas and malignant carcinomas. Using the plant-derived alkaloid cyclopamine as an inhibitor of HH signalling, we found a dose-dependent decrease in cell yield with a concomitant induction of apoptosis in these cell lines [6]. We were able to rescue the cyclopamine-induced apoptosis by adding an excess of SHH ligand. These data suggested that hedgehog signalling has a tumour-promoting effect on cell survival in both benign and malignant colorectal tumours.

Since our initial observations were published, the role of hedgehog signalling in CRC has been somewhat controversial, with published studies failing to reach agreement for several years on the presence and activity of the pathway in colorectal tumour cells, or indeed the consequences of pathway activation. Studies in a variety of experimental systems now point towards a consensus on the presence HH in neoplastic colorectal epithelium: Oniscu *et al.* [7] reported expression of SHH, PTCH and SMOH in hyperplastic colorectal polyps, adenomas and carcinoma, and also showed increased cell growth in primary mouse colonocytes treated with SHH peptide. Douard *et al.* [8] found upregulation of SHH in the colorectal tumours of 38 out of 44 patients, when compared with matched normal tissue, and that SHH increased proliferation in the CRC cell line HT29. The data from these two studies concurred with our initial findings of autocrine HH activity in CRC [6].

More recently, a study reported that knocking down SMOH in human CRC cell lines suppressed cell proliferation, and that SMOH expression was increased in polyps from a mouse model of colorectal tumorigenesis with mutant *APC* [9]. A study by Varnat *et al.* [10], using combined *in vitro* and *in vivo* approaches, showed HH signalling to be essential for human colon cancer cell growth, and also for metastasis in a xenograft model. Small molecule inhibition of GLI1 and GLI2 has been shown to induce cell death in colorectal carcinoma cell lines [11]. Whereas Yoshimoto *et al.* [12] showed decreased cell viability and BrdU incorporation in HT29 cells following HH blockade, suggesting a HH-dependent suppression of apoptosis, and modulation of localised immune response through cytokine regulation. Taken together, these studies also support our initial report of inducing apoptosis in colorectal tumour cells by inhibiting the HH pathway with cyclopamine [6].

During embryological development and adult tissue homeostasis, the HH pathway frequently acts in concert with other key signalling pathways, including the Wnt family of signalling proteins. The role of the Wnt signalling pathway in both the genesis and malignant progression of colorectal tumours is well documented, with mutations resulting in pathway activation in the vast majority of cases [2]. However, although there are published reports of HH-Wnt interaction in CRC, the nature and consequence of this relationship is not yet clear.

A study by van den Brink *et al.* [13] showed that signalling via IHH antagonised Wnt signalling in the colonic crypt *in vivo* and also CRC cells *in vitro*. Other work on gastric adenocarcinoma cell lines showed HH to induce the Wnt inhibitor sFRP-1, but as this acts upstream of the APC-mediated β-catenin destruction complex (disrupted in the vast majority of CRCs) is unlikely to be of consequence in colorectal tumours [14]. However, counter to these reports, SMO knockdown in CRC cell lines has been shown to suppress β-catenin-dependent transcription [9].

Furthermore, the reported HH antagonism of Wnt does not appear to be reciprocal. Maeda and colleagues [15], showed that β-catenin enhanced GLI1-dependent transcriptional activity in lung and gastric cell lines, but also in the CRC cell line SW480. The authors suggested that β-catenin may play an integral part in the HH pathway [15].

Having previously shown that the hedgehog inhibitor cyclopamine can reduce cell yield and induce apoptosis in both benign and malignant colorectal tumour cells [6], and knowing the importance of Wnt signalling in CRC survival we were curious to discover whether cyclopamine could affect Wnt signalling, already defective in these cells. To this end we measured the response of catenin-related transcription (using the TOPFLASH Tcf-reporter assay [16]) to cyclopamine with or without the addition of exogenous amino terminal SHH peptide.

## 2. Results and Discussion

### 2.1. Cyclopamine Treatment Reduces β-Catenin-Related Transcription in the Colorectal Cancer Cell Line SW480 in a Dose-Dependent Manner

**Figure 1:**
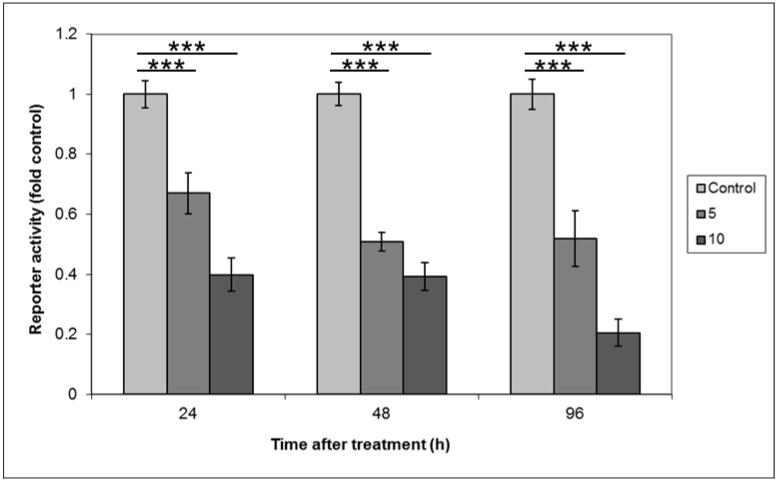
β-catenin related transcription is reduced in response to cyclopamine treatment. SW480 cells were treated with 5 or 10 µM cyclopamine for 24, 48, or 96 h. Data shows the activity of the TOPFLASH reporter, corrected using FOPFLASH, and normalised to the *Renilla* control plasmid. The data represents the mean from three separate experiments performed in triplicate and the values shown are fold control. Statistical analysis was performed using the Student’s *t*-test, where degrees of significance are indicated as * *p* < 0.05, ** *p* < 0.01, *** *p* < 0.001 and the error bars represent SEM.

In order to ascertain the potential effect of HH signalling on Wnt signalling output, the HH pathway was inhibited in the SW480 cell line by treatment with cyclopamine, compared with vehicle control. Samples were harvested after 24, 48 and 96 h of treatment. β-catenin-related transcription (CRT) was measured using the TOPFLASH reporter assay [16], and the data was corrected using the FOPFLASH control vector. The data from three independent experiments performed in triplicate is shown in Figure 1.

**Figure 2:**
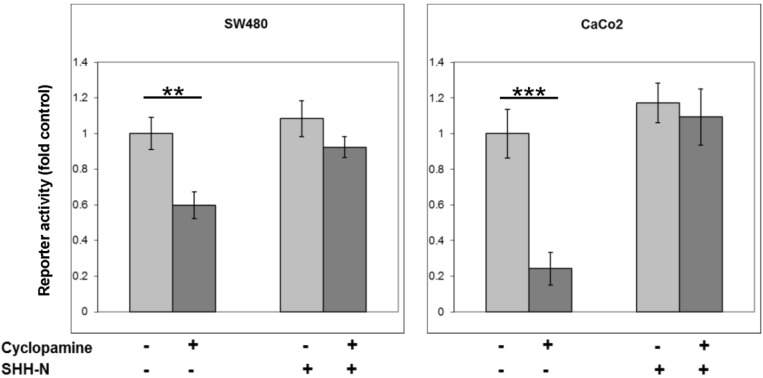
The inhibitory effect of cyclopamine on β-catenin-related transcription can be reversed by exogenous addition of SHH-N. SW480 or CaCo_2_ cells were treated with either 5 µM cyclopamine, SHH-conditioned medium, or both for 48 h. Data shows the activity of the TOPFLASH reporter, corrected using FOPFLASH, and normalised to the *Renilla* control plasmid. The data represents the mean from three separate experiments performed in triplicate and the values shown are fold control. Statistical analysis was performed using the Student’s *t*-test, where degrees of significance are indicated as * *p* < 0.05, ** *p* < 0.01, *** *p* < 0.001 and the error bars represent SEM.

These data clearly show a robust and statistically significant (*p* ≤ 0.001) inhibition of CRT in SW480 cells by cyclopamine that is not only dose-dependent, but also sustained over the 96 h of the experiment. In order to show that this inhibition of canonical Wnt signalling by cyclopamine was due to inhibition of HH signals, and not due to potential off-target effects of the drug, recombinant SHH was added back into the system. Active amino terminal SHH (SHH-N) was produced by transient transfection of HEK293 cells as previously described [17]. Medium conditioned with SHH-N was then used to treat SW480 or CaCo2 cells, with or without the addition of cyclopamine, and the TOPFLASH reporter assay performed. The data from three independent repeats of this experiment (in triplicate) are shown in Figure 2.

The addition of SHH-N alone produced a modest, but not statistically significant, elevation in CRT activity. This increase should be considered in the context of abnormally high CRT in these cells due to mutations in the Wnt pathway. However, SHH-N in combination with cyclopamine, was also able to reverse the effect of the drug on CRT in CaCo2 and SW480 cells, demonstrating that the effect of cyclopamine on CRT was due to inhibition of signalling through the HH pathway.

These data suggest a positive synergy between HH and Wnt signalling in terms of regulating CRT in CRC cells. This is the first time that cyclopamine has been shown to have this effect in this model, but this finding does fit with the observations of others. Maeda *et al.* [15] showed that β-catenin was able to enhance the transcriptional activity of Gli1, whereas our data show a potential reciprocation in terms of HH enhancing Wnt output. The findings of Arimura *et al.* [9] concur with this, reporting that SMOH was “unexpectedly” able to positively regulate nuclear β-catenin levels in SW480 and HCT116 cells. The demonstration here of a combined inhibition of two pathways known to be vital in CRC development and progression by a drug (cyclopamine) highlights therapeutic potential, and has implications for our understanding of the role of combined signalling in colorectal cancer pathology.

These findings may appear to contradict earlier studies showing antagonism between the Wnt and HH pathways. However, van den Brink *et al.* [13] showed that it was Indian hedgehog (IHH), and not Sonic (SHH), that affected Wnt signalling, and this was observed in the context of butyrate-induced cellular differentiation. Furthermore, Fu *et al.* [18] showed that IHH expression negatively correlated with *APC* mutations in colorectal tumours both *in vivo* and *in vitro*, yet Büller and colleagues [19] showed that stromal IHH was required for adenoma formation in a mouse model, albeit through a paracrine effect on the tumour stroma.

These data show that we do not yet fully understand the role of HH in CRC and raises the question of the potential for distinct roles for SHH and IHH in CRC cells, perhaps acting in a highly context-dependent manner. Our initial study of HH expression in cell lines derived from benign colonic adenomas showed relatively high levels of IHH and lower levels of SHH, whereas the reverse was true in carcinoma cells [6].

The idea of a temporal separation of HH signalling between the benign and malignant stages of colorectal tumours, and a switch from the state in normal tissue, is further highlighted by Varnat *et al.*’s [10] finding that SHH was involved in metastatic progression. Tumour invasion being the defining difference between benign and malignant colorectal tumours.

Further investigation is needed in order to enhance our understanding of the complex integration of these signals in the context of the strong, yet complex data published in the studies described.

### 2.2. Cyclopamine Treatment Induces the Expression of E-Cadherin in Both Benign and Malignant Colorectal Tumour-Derived Cell Lines and Reduces Invasion in SW480 Cells

The metastatic process in CRC has been shown to involve a phenomenon known as the epithelial-mesenchymal transition (EMT) [3,20]. A key feature of this process is the loss of expression of the cell-cell adhesion molecule E-cadherin, whose loss correlates with poor prognosis in CRC patients [20]. Both the Wnt and HH signalling pathways have been implicated in the regulation of EMT in both normal tissues, and in cancers [21]. As we now show that cyclopamine can reduce the output of both HH and Wnt pathways, the effect of cyclopamine was measured on E-cadherin expression and the invasive capability of CRC cells.

**Figure 3:**
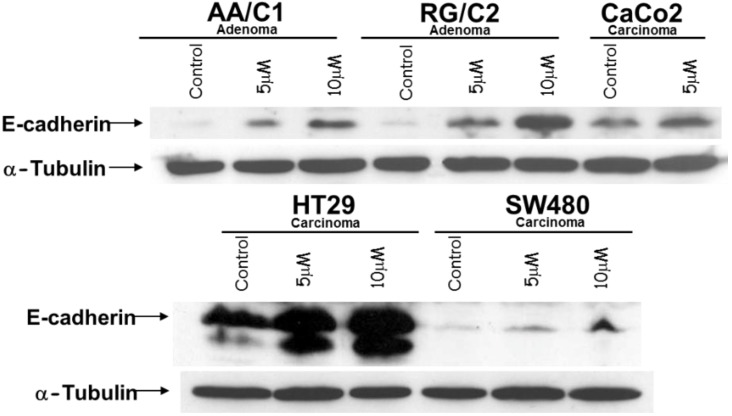
Cyclopamine treatment induces E-cadherin expression in colorectal tumour cells. Adenoma or carcinoma-derived cells were treated with 5 or 10 µM cyclopamine 48 h prior to lysis. Western blots were performed for E-cadherin, using α-tubulin as a control to show equal sample loading. Data shown are representative of three separate experiments. The lower blot shown has been overexposed to show induced expression in SW480 relative to HT29. The 10 µM dose was not used on CaCo_2_ cells due to their cyclopamine sensitivity described previously [6].

**Figure 4:**
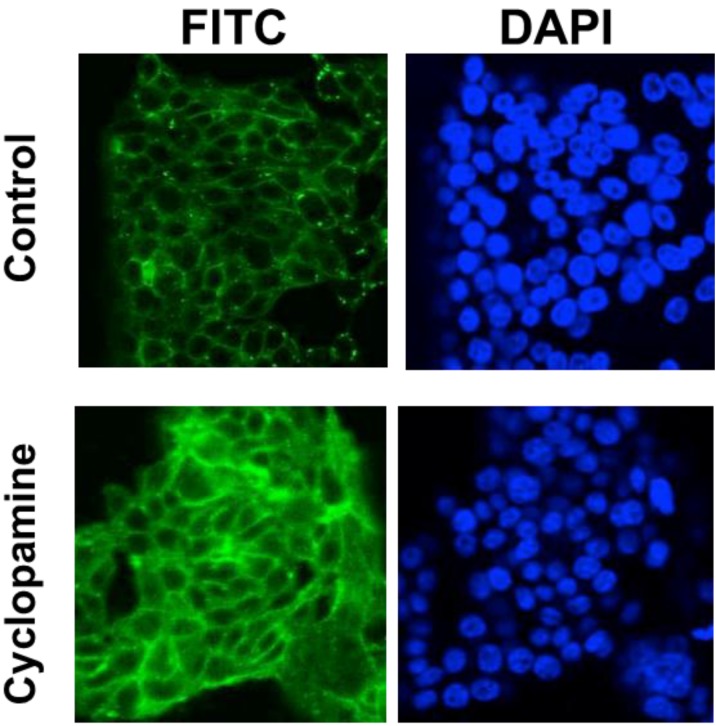
Cyclopamine treatment induces E-cadherin expression in colorectal tumour cells. HT29 cells were treated with 10 µM cyclopamine 48 h prior to fixation and immunofluorescent labelling for E-cadherin. Data shown are representative of three separate experiments.

A panel of cell lines derived from both colorectal adenomas and carcinomas were treated with cyclopamine for 48 h and then lysed for western blotting analysis. The results of these analyses are shown in Figure 3. E-cadherin expression was induced by cyclopamine in each of the cell lines tested, including SW480, which has relatively low levels of the protein. The level of expression relative to HT29 is illustrated in the lower blot shown, which has been overexposed to show induced expression in SW480. E-cadherin facilitates cell-cell adhesion by forming adherens junctions between adjacent cells. This means that functionally active E-cadherin will be found at the cell membrane where the cell is in contact with its epithelial neighbours. Immunofluorescent staining was carried out in order to determine the subcellular localisation of E-cadherin following cyclopamine treatment in CRC cells. Representative results of this analysis are shown in Figure 4. The cyclopamine-treated cells show a stronger, and more distinct membranous pattern of E-cadherin expression. This finding is suggestive that the upregulation of E-cadherin observed following cyclopamine treatment is resulting in the formation of adherens junctions and therefore increased cell-cell adhesion.

The ability of cyclopamine to induce E-cadherin is significant, especially as this effect was observed in cells derived from benign adenomas as well as invasive carcinomas. As this would indicate an inhibition of malignant transformation and metastatic potential, E-cadherin induction was even found in SW480, which has very low levels of E-cadherin and provides an *in vitro* model of EMT [22].

**Figure 5:**
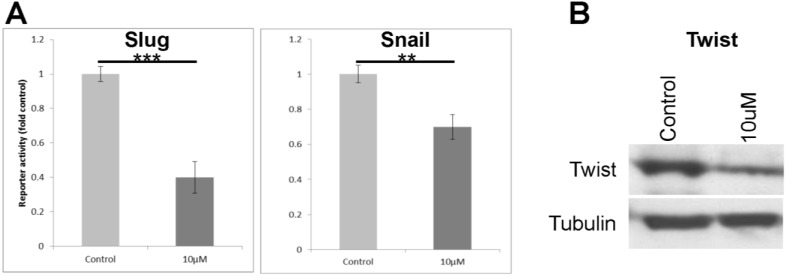
Cyclopamine treatment reduces the promoter activity of the Snail and Slug gene promoters and also of the expression of Twist protein in SW480 cells. (**A**) SW480 cells were treated with 10 µM cyclopamine for 48 h having been previously transfected with the Slug or Snail promoter reporter constructs. Data shows the activity of the Slug or Snail reporters, corrected using empty vector controls, and normalised to the *Renilla* control plasmid. The data represents the mean from three separate experiments performed in triplicate and the values shown are fold untreated control. Statistical analysis was performed using the Student’s *t*-test, where degrees of significance are indicated as * *p* < 0.05, ** *p* < 0.01, *** *p* < 0.001 and the error bars represent SEM; (**B**) SW480 cells were treated with 10 µM cyclopamine for 48 h and then lysed for western blot analysis (for Twist). The western blot analysis is representative of three separate experiments.

Downregulation of E-cadherin expression in tumours is associated with EMT. Several transcription factors have been implicated as key mediators of EMT, including slug, snail and twist (reviewed by Gonzalez and Medici [21]). The response of these transcription factors to cyclopamine treatment was tested in SW480 cells. Figure 5 shows the results of promoter-reporter analysis for *Slug* and *Snail*, and also a western blot for Twist. Cyclopamine treatment leads to a down regulation of all three EMT-associated transcription factors, further highlighting the potential of this drug as an inhibitor of EMT and therefore malignant progression in colorectal tumours.

**Figure 6:**
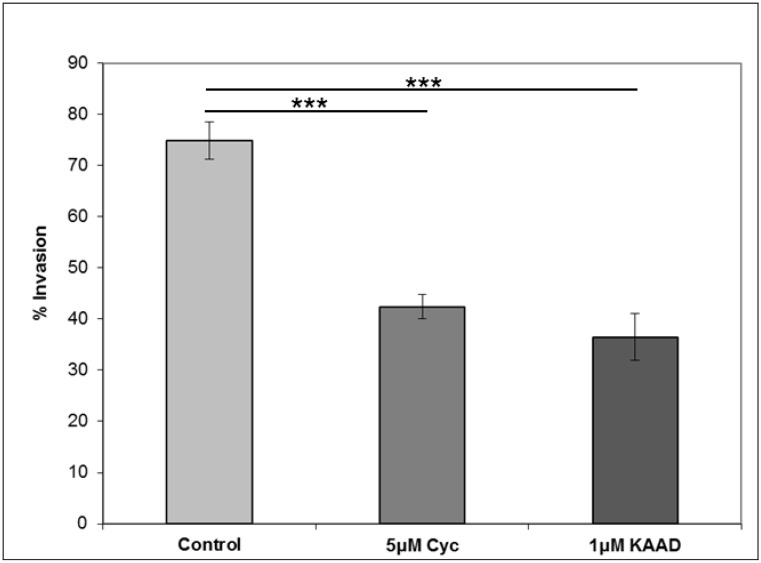
Cyclopamine reduces invasion in SW480 cells. SW480 cells were treated with 5 µM cyclopamine, 1 µM KAAD-cyclopamine, or vehicle control for 24 h whilst growing on a Matrigel™-coated transwell filter. Data shown are expressed as percentage invasion and represent the mean of three separate experiments performed in triplicate. Statistical analysis was performed using the Student’s *t*-test, where degrees of significance are indicated as * *p* < 0.05, ** *p* < 0.01, *** *p* < 0.001 and the error bars represent SEM.

E-cadherin expression inversely correlates with invasive potential in epithelial tumours by holding the cells together. Downregulation of E-cadherin during EMT allows tumour cells to detach from the tumour mass, invade the underlying tissue and eventually go on to form lethal distant metastases. In order to test whether cyclopamine could influence invasion in colorectal cancer cells, a Matrigel™-coated transwell filter assay was performed. Figure 6 shows that both cyclopamine and KAAD-cyclopamine are able to significantly (*p* ≤ 0.001) inhibit the invasion of SW480 cells into Matrigel™.

These data, combined with the findings described above, suggest that cyclopamine, or drugs with a similar mode of action, could have a therapeutic benefit in the treatment of CRC. Other studies have also indicated that HH can affect invasion and metastasis in other tumour types: Gao *et al.* [23] showed HH inhibition to reduce invasion in an *in vitro* model of gastric carcinoma; whereas Ke *et al.* [24] showed the SHH-Gli1 axis to drive EMT in ovarian cancer cells. Similarly, Huo *et al.* [25] showed HH to promote EMT, invasion and metastasis in both *in vitro* and *in vivo* models of non-small cell lung cancer, and were also able to abrogate these effects with cyclopamine. Varnat *et al.* [10,26] implicated HH signalling, through interaction with Wnt, to be involved in the metastatic progression of colorectal tumours, which supports the data described above on the effects of cyclopamine treatment.

## 3. Experimental Section

### 3.1. Cell Culture and Cyclopamine Treatment

The colon carcinoma derived cell lines CaCo2, HT29, and, SW480 were obtained from the ATCC and were cultured in DMEM supplemented with 10% (*v*/*v*) FBS as adherent monolayers in 25 cm^2^ tissue culture flasks. AA/C1 is a clonogenic, adenoma cell line derived from a 3–4 cm polyp from the descending colon of a familial adenomatous polyposis (FAP) patient [27] and is cultured in conditioned medium as described by Williams *et al.* [28]. RG/C2 is a clonogenic cell line derived from a sporadic tubular adenoma of the sigmoid colon of 1–2 cm in diameter and is cultured in DMEM supplemented with 20% (*v*/*v*) FBS [29]. Both of these adenoma-derived cell lines are anchorage-dependent and are non-tumorigenic in athymic nude mice.

Cyclopamine (Toronto Research Chemicals) was dissolved in 95% ethanol. To minimise the effects of growth factors present in serum, cyclopamine treatments were carried out using DME-F12 medium (Invitrogen) supplemented with 2% FBS as described previously [6]. This medium is hereafter referred to as “reduced serum medium” (RSM). Corresponding vehicle controls were prepared by the addition of 0.1% (*v*/*v*) of 95% ethanol. Initial experiments, carried out with 0.1% (*v*/*v*) ethanol showed no negative effect on cell growth in any of the cell lines used.

### 3.2. Promoter-Reporter Assays

For the measurement of β-catenin-related transcription, the plasmids pTOPFLASH and pFOPFLASH [16] were obtained from Upstate Biotechnology (NY) and were used as described previously [30]. Cells were seeded in six well plates at 8 × 10^5^ cells per well. After 48 h growth, cells were transfected with 1.8 µg of TOPFLASH or FOPFLASH along with 0.2 µg of CMV-*Renilla* control plasmid (Promega, Madison, WI, USA) using Superfect transfection reagent (Qiagen, Valencia, CA, USA).

The *Slug* and *Snail* promoter-reporter plasmids were a kind gift from Antonio de Herreros (UPF, Barcelona) and were used as described above. Reporter activity was measured on a Jade luminometer (Labtech, East Sussex, UK) using the Dual Luciferase reporter assay system (Promega). Samples were corrected for background using untransfected cells as a control.

### 3.3. Production of Recombinant SHH-N Protein

The pSHH-N plasmid was a kind gift from Dr Phil Beachy (Baltimore). Active amino-terminal SHH (SHH-N) was produced by the transient transfection of HEK293 cells as described previously [6,17] and the medium assayed for SHH content by Western blotting.

### 3.4. Western Blot Analysis

Samples of 1 × 10^6^ cells were prepared for western blotting as described previously [6]. Antisera toE-cadherin was obtained from BD Transduction Labs (CA) and for Twist from Santa Cruz Biotech (CA). Blots were subsequently probed with anti α-tubulin (Sigma, Gillingham, UK) to show equal sample loading.

### 3.5. Immunocytochemistry

Cells were seeded onto chamber slides (Nunc, Naperville, IL, USA). Following 48 h of growth, cells were treated accordingly. The cells were then fixed and permeabilised in 4% formaldehyde containing 0.2% TRITON X-100 for 10 min. Fixed slides were washed in PBS, and then blocked in PBS containing 2% BSA and 5% FBS. The primary antibody for E-cadherin (BD Transduction Labs (CA)) was diluted in block, applied and incubated for 1 h. Following 5 washes in PBS and 1 in block, slides were incubated in FITC-conjugated secondary antibody for 1 h. Following 5 washes in PBS and 2 in distilled water, coverslips were mounted in Vectashield with DAPI (1.5 µg/mL, Vector, San Diego, CA, USA). Specimens were viewed on a Leica TCS-NT confocal laser scanning microscope and images captured using Leica TCS-NT software. In order to determine background levels of non-specific fluorescence, cells were stained with only the FITC-conjugated second antibody and viewed as above.

### 3.6. In Vitro Invasion Assay

Cell invasion assays were carried out using a 6 well plate transwell filter migration assay as previously described [31]. For quantitative analysis of cell invasion 8 μm pore size transwell filters pre-coated with Matrigel™ (Becton Dickinson, UK) were used. 1 × 10^5^ cells were seeded per well in calcium-free DMEM containing 0.1% FBS (CF-DMEM) and allowed to adhere for four hours. Cells were then treated with cyclopamine or vehicle at the doses indicated in CF-DMEM for 24 h. After the 24-h incubation cells were removed from the upper filter surface with a cotton swab. The filters were fixed and stained with haematoxylin. Cells in the lower filter surface were considered invasive and counted in 10 fields at ×200 magnification. Three independent experiments were carried out in triplicate, and the data are expressed as the mean ± S.E.M. Statistical analysis of this data was performed using the Student’s *t*-test. Differences were considered significance when the *p* value was <0.05.

## 4. Conclusions

It has been estimated that around 90% of all cancer deaths are caused by metastasis, and around half of all patients presenting with CRC have advanced disease when presenting at the clinic [32,33]. Although prevention and early detection could save many lives, there is a pressing need to further our understanding of the acquisition of a metastatic phenotype in CRC cells.

The importance of Wnt signalling in CRC is well established, but the emerging importance of HH has been less clear-cut despite some excellent work in the area. Unlike Wnt, the HH pathway appears to be rarely mutated, and it seems that it may become activated due to a switch in the signalling context. Several studies point towards a switch from the paracrine role of HH in classical epithelial-mesenchymal interactions, towards an autocrine role in cells that have become self-sufficient in Wnt (through loss of APC function), and therefore freed from the regulatory powers of their mesenchymal neighbours.

Some researchers suggest that autocrine HH signalling may be indicative of a more stem cell like phenotype. Batsaikhan *et al.* [34] showed that cyclopamine reduced stem cell markers in the CRC cell line HCT116, and Varnat *et al.* [26] showed that stem cell self-renewal relied on HH-GLI activity *in vivo*. Further work is required to more fully understand the potential role of HH, now in the context of Wnt activation, on maintenance of CRC stem cells.

Although HH and Wnt signalling are known to act together in many tissues at various times during embryological development, the crosstalk between the pathways in CRC has proved rather more difficult to elucidate. Seemingly contradictory studies have been published, but often using different model systems (both *in vivo* and *in vitro*), and the nature of these pathways means that any response is likely to be highly dependent on the precise conditions and context of the model used.

The molecular basis of crosstalk between HH and Wnt is becoming increasingly well understood (reviewed in [35,36]). Varnat *et al.* [37] suggest that HH signalling may act in parallel, or downstream of Wnt. However, Yoshimoto *et al.* [12] reported that exposing the CRC cell line HT29 to recombinant SHH resulted in the nuclear exit and membrane accumulation of β-catenin (consistent with its role in forming adherens junctions). These investigators did not, however, report the effect of SHH on CRT in this model. Conversely, and in close agreement with our own data, Arimura *et al.* [12] showed that siRNA knockdown of *SMO* in CRC cell lines reduced CRT. In the current study we have endeavoured to focus on the action of the drug cyclopamine and the measurement of biological endpoints directly relevant to the clinical management of colorectal cancer.

HH signalling is known to have a role in the regulation of non-pathogenic EMT (reviewed by Gonzalez and Medici [21]) and our understanding of this is broadening. For example, Tang *et al.* [38] have recently shown that signalling via Gli1 and Gli2 is required for EMT in human trophoblasts during placental development.

EMT has also become established as a phenomenon important for metastatic tumour progression. HH signalling has recently become implicated in regulating this process in a number of cancer types, including gastric, lung and ovarian carcinomas [23,24,25].

We have now shown strong evidence for cyclopamine to reverse EMT in CRC cells. It remains to be shown whether this is due to effects on HH directly, or it’s crosstalk with Wnt signalling. This being the key question for our future investigations. Varnat *et al.* [26] have shown that HH promotes metastatic spread in an *in vivo* model of CRC and postulate that metastatic potential is achieved in CRC cells upon acquisition of a primitive ES-like stem cell phenotype. This finding should also inform future studies into HH and CRC metastasis. Finally, work on ovarian cancer cells by Ke *et al.* [24] has demonstrated that SHH drives EMT via the PI3K/AKT pathway, which presents itself as worthy of investigation as a potential mechanism in CRC.

Our final finding, that cyclopamine inhibits tumour cell invasion in an *in vitro* model represents the most clinically significant observation that we report. The acquisition of the invasive phenotype is essential for metastasis and treatments targeting this process have great potential in treating patients with advanced disease. In a similar approach, Gao *et al.* [23] showed HH inhibition to reduce invasion in an *in vitro* model of gastric carcinoma, and as described above, Varnat *et al.* [26] reported HH to promote metastasis in a mouse model. Both of these findings suggest that our data not only represent a significant finding in CRC, but a mechanism that may be of importance in other epithelial tumour types as well.

In conclusion, as Wnt signalling is known to be the key driving force in colorectal tumorigenesis and it is, therefore, significant that we have been able to demonstrate a pharmacological inhibition of this pathway. The ability to upregulate E-cadherin has potential clinical significance due to the correlation between its expression and prognosis. The data we show suggests that this reconstitution of E-cadherin is part of a mesenchymal-epithelial transition, and therefore represents a potential target for inhibiting the metastatic potential of colorectal tumours, reinforced by the ability of cyclopamine to reduce tumour cell invasion in an *in vitro* model.
